# From the hospital to the community: reshaping the palliative care paradigm

**DOI:** 10.3389/fpubh.2026.1876918

**Published:** 2026-07-09

**Authors:** Carlos Laranjeira, Ana Ramos, Gisele Reami, Ana Querido

**Affiliations:** 1 School of Health Sciences of Polytechnic of Leiria, Leiria, Portugal; 2 Center for Innovative Care and Health Technology (ciTechCare), Polytechnic of Leiria, Leiria, Portugal; 3 Comprehensive Health Research Centre (CHRC), University of Évora, Évora, Portugal; 4 RISE-Health, Nursing School of Porto (ESEP), Porto, Portugal

**Keywords:** care transition, community, compassionate movement, palliative care, person-centered approach

## Abstract

Palliative care (PC) has traditionally developed within hospital settings, shaped by biomedical models, specialist hierarchies, and episodic care delivery. However, demographic change, rising multimorbidity, patient preferences, and health system constraints increasingly challenge hospital-centric approaches. In contrast, improving the community palliative care model requires moving beyond the simple decentralization of services toward a more integrated, person-centered, and ethically grounded system of care. At its core, an effective community model must recognize the individuality of people living with serious illness and respond to their needs across time, relationships, and care settings rather than in isolated clinical encounters. This perspective paper argues that a paradigm shift from hospital-based to community-anchored PC is no mere organizational adjustment but requires a conceptual transformation. Drawing on recent evidence and global policy frameworks, the paper explores the drivers, opportunities, and tensions of community-based PC, highlighting implications for clinical practice, professional roles, health systems, and communities. The transition between models is framed as a reorientation toward continuity, relational care, and shared responsibility, requiring new forms of integration between specialist services, primary care, and civil society.

## 1 Introduction

According to the Organization for Economic Co-operation and Development's report (1), despite the gradual recovery of countries after the pandemic, there are persistent structural challenges in health systems around the world. Heart disease and cancer accounted for nearly half of deaths, with more than 3 million preventable premature deaths recorded among people aged under 75 years. Among primary healthcare users aged 45 and over, 82% reported at least one chronic disease and 52% reported two or more (1). It is estimated that each year, 56.8 million people, including 25.7 million terminally ill, need PC. Worldwide, only about 14% of people who need PC currently receive it (2). PC aims to alleviate physical, psychosocial, and spiritual suffering across the life course, and has emerged as a cornerstone of quality health care for people living with serious illnesses and for their informal caregivers (3). Historically, its development has been closely linked to hospices, hospitals and specialist services, where expertise, technology, and acute care infrastructures are concentrated. While this model has delivered undeniable benefits, it now appears increasingly misaligned with population needs and preferences.

Most people with life-limiting illness spend most of their illness trajectory in the community and consistently report a preference to remain—and often to die—at home rather than in hospital (4). Yet health systems continue to default to hospital-based responses, particularly in moments of crisis. This mismatch has contributed to avoidable hospital admissions, fragmented care, caregiver distress, and escalating costs (5). Recently, policymakers and scholars have given greater attention to community-based and primary PC models, signaling a broader paradigm shift toward approaches that are delivered within communities, owned by communities, and shaped through active community participation (6, 7).

The structure of this perspective paper was shaped by a purposive literature search, which offered a critical perspective on what it means to move PC “from the hospital to the community”. Searches were conducted in major databases (including PubMed, CINAHL, and Scopus), using combinations of terms such as ‘palliative care', ‘community-based care', ‘transitional care', ‘integrated care', and ‘compassionate communities'. Priority was given to literature published in the last 5-10 years, alongside seminal works, to ensure both contemporary relevance and conceptual grounding. Sources were selected based on their relevance, methodological rigor, and contribution to theoretical and policy debates. Through this approach, we argue that a genuine shift entails deep, multi-faceted changes in how care is organized and delivered, how responsibility is shared among formal and informal actors, and how expertise is recognized and mobilized across settings.

## 2 Limitations of the hospital-centric palliative care model

In 2024, health expenditure represented a significant share of the economy and public spending in OECD countries (1); however, this high level of investment does not necessarily ensure financial protection, as many households still face catastrophic health expenditure due to out-of-pocket costs (8). Projections indicate that this investment is likely to increase in the coming decades, driven by population aging, technological advancements, and growing expectations regarding the responsiveness of healthcare systems. In 2023, prevention and primary healthcare represented a relatively small share of total expenditure, despite their central role in healthcare organization. Still, primary care contributed to the reduction of avoidable hospitalizations in 28 of the 30 OECD countries, and 87% of users with chronic diseases positively evaluated the primary care received (1). Population aging and the increasing prevalence of chronic conditions contribute to the growing number of people needing end of life (EoL) care; this number is estimated to reach 10 million by 2050 (9). EoL care can be provided in hospitals, hospices, long-term care facilities, or at home. The preferred place of care is shaped by personal and cultural factors and may change over time, but the literature reports a general preference for home at the EoL, while acknowledging significant variability and methodological limitations in preference research (10–12).

While the OECD's vision of “Time for better care at EoL” promotes integrated, equitable, and person-centered palliative care systems, its translation into practice reveals important divergences between the Global North and Global South (9). In high-income settings, challenges persist in overcoming institutional inertia, social inequalities, and fragmented care delivery, whereas in low- and middle-income contexts, policy aspirations are often constrained by limited resources, high out-of-pocket expenditure, and a heavy reliance on informal and community-based care, resulting in substantial unmet needs (13).

As health systems operate with finite resources, it is essential to identify patients with greater clinical complexity to allocate resources where they can generate greater benefit. The diversity of needs and preferences requires systematic assessment to ensure adequate access to services. In this context, primary and general care, due to their proximity and continuity of care, assume a central role in the provision of quality PC. They can be mobilized more strategically when supported by a structured identification of PC needs, thereby promoting adequacy, continuity, and better management of available resources (14).

Hospital-based PC evolved in response to unmet symptom control and communication needs within acute care settings. However, with growing demand, this model's limitations have become increasingly evident. Hospitals are designed for episodic, intervention-focused care and are poorly suited to the longitudinal, relational, and socially embedded nature of palliative needs.

Transitions from hospital to home care are widely recognized as vulnerable stages. Evidence suggests that even when inpatient PC improves symptom burden and discharge planning, the outcomes following hospital discharge remain inconsistent, with high risks of readmission and discontinuity of care (15). These challenges reflect structural fragmentation between hospital and community services rather than failures of individual clinicians (16, 17). Moreover, hospital-centric models often reinforce late referral patterns, positioning PC as an EoL intervention rather than an early and integrated approach. This contributes to inequitable access (particularly for people with non-cancer diagnoses), frailty, or social vulnerability for those with limited hospital contact (18, 19).

## 3 Community-based palliative care

Allan Kellehear's work underpins the link between a public health approach to palliative care and compassionate communities by emphasizing that dying, death, and bereavement are social experiences, not just medical events (20). He argues that most EoL care is provided by informal networks, so improving care requires strengthening the community's capacity alongside health services. Drawing on health promotion principles, Kellehear encourages “death literacy” and community engagement, leading to the idea of compassionate communities—where care becomes a shared civic responsibility, integrating social support with professional palliative care (21).

Community-based PC encompasses home-based care, outpatient clinics, primary care–led models, and integrated specialist–generalist partnerships. A substantial body of evidence now supports the clinical, experiential, and system-level benefits of these approaches. The literature consistently demonstrates that home-based and community PC improves quality of life, reduces hospital admissions and length of stay, and increases the likelihood of dying at home in accordance with the person's preferences (22).

Cost analyses indicate that community care may be associated with higher outpatient resource use, but overall healthcare costs may be reduced when hospital use decreases (23, 24). Evidence also indicates that the most effective community models are those characterized by continuity of care, interdisciplinary teamwork, proactive care planning, and strong integration with primary health care. Importantly, no single model fits all contexts; effectiveness depends on adaptation to local resources, population needs, and health system structures (25, 26).

Caregivers' motivations for providing care at home are related to factors such as the nature of their relationship and bond with the patient; their personal traits motivating them to provide care; and social expectations or filial obligations (27). In contexts marked by a family-centered culture, the primary responsibility for care falls on the family. These tensions are exacerbated by regional inequalities in access to community-based PC teams and the absence of standardized protocols for the transition between hospital and home. As a result, families assume an active role in the continuity of care, often without receiving adequate support during this transition between institutional and home care (28).

Several countries and organizations have developed structures and intervention models aimed at expanding access to and improving the quality of PC, including strategies focused on the transition from hospital to home at the EoL (29). Transition of PC refers to the transfer of patients between different services or care settings, with the aim of ensuring continuity of care. When these transitions are not carried out properly, they can result in worse clinical outcomes and greater use of health services. A synthesis of 16 randomized studies (*n* = 3,781) indicates that transitional care interventions within a PC approach have no clear effects on mortality, functional status, or caregiver burden compared with standard care. Findings vary and are limited methodologically, providing low to very low certainty evidence of modest improvements in patients' quality of life and symptom burden (30). In this context, promoting safe and effective transitions requires stakeholder involvement, a stronger evidence base, the development of specific policies, and the integration of PC into health settings not traditionally oriented toward this approach (31).

Recent studies on the transition of PC from hospital to home highlight several challenges for informal caregivers (28, 32). Among the most frequently reported aspects are the administrative overload associated with organizing hospital discharge, difficulties in communication and coordination between health teams, and the need for caregivers themselves to manage essential support, such as equipment and medical supplies. After returning home, many caregivers report feeling unprepared to handle complex caregiving responsibilities (often due to lack of formal guidance or structured support). This can lead to anxiety, insecurity, and fear of making mistakes. Furthermore, a discrepancy is frequently observed between caregivers' expectations and the actual experience of home care, particularly regarding the availability of services and the number of hours of home support (29). The accumulation of these responsibilities contributes to high levels of physical and emotional exhaustion and feelings of isolation. Nevertheless, the presence and availability of community-based PC teams are described as an important support factor, providing guidance, emotional validation, and a greater sense of security during adaptation to home care (28, 33).

The shift to PC in the community challenges traditional professional boundaries. Specialist teams alone cannot meet growing demand; instead, generalist providers—particularly general practitioners and community nurses—must play central roles in delivering primary PC. This redistribution of responsibility requires a reconceptualization of expertise, moving from ownership of care to facilitation, mentorship, and shared decision-making. Evidence suggests that integrated models, where specialists support primary care teams through joint visits, advice lines, and education, can improve confidence, coordination, and patient outcomes. However, role ambiguity, time constraints, and limited training remain persistent barriers (26, 34). Such changes also have ethical implications. A community-based paradigm emphasizes relational continuity, moral responsibility, and proximity to the patient's lived contexts. It demands that PC be understood not only as a clinical specialty but as a core competency across health and social care (6, 35).

## 4 Communities as care partners: beyond services

Perhaps the most radical dimension of this change in strategy lies in the repositioning of communities themselves. Public health and compassionate community approaches emphasize that care for people who are dying cannot be fully professionalized and must involve families, neighbors, volunteers, and social networks. Research highlights that strong community support networks can prevent unnecessary hospitalizations, improve caregiver wellbeing, and foster more humane EoL experiences. During and after the COVID-19 pandemic, the resilience—or fragility—of these networks was particularly visible (18, 36).

The growing recognition that PC cannot remain anchored primarily in hospitals signals more than a service redesign; it reflects a fundamental rethinking of how societies care for people who are facing serious illness, dying, and loss. As health systems grapple with aging populations, multimorbidity, workforce shortages, and escalating costs, the hospital-centric PC paradigm appears increasingly unsustainable (37). Yet the move “from hospital to community” should not be understood as a simple transfer of location. Instead, it represents a strategic shift whereby communities are repositioned as active care partners rather than peripheral backgrounds to professional care.

At its core, PC addresses suffering that is deeply embedded in everyday life: symptoms are experienced at home, uncertainty unfolds within families, and meaning is negotiated in social and cultural contexts. Hospitals, while indispensable for acute and complex interventions, are ill-equipped to respond to these relational and longitudinal dimensions of care. Over-medicalization at the EoL often disrupts continuity, distances people from their social worlds, and transforms dying into a sequence of clinical events (38). The community, by contrast, is where lives are lived, identities maintained, and relationships sustained. Recognizing communities as care partners therefore aligns PC with the realities of patients' lives rather than the convenience of healthcare institutions.

Communities contribute to PC first and foremost through informal care networks. Family members, friends, neighbors, and volunteers already provide most of the day-to-day care for people with life-limiting illnesses, frequently without recognition, preparation, or support (39). A community-oriented paradigm does not romanticize this care, nor does it seek to replace professional services. Instead, it recognizes and highlights this existing infrastructure and seeks to strengthen it through education, coordination, and professional backup. When communities are engaged deliberately, caregiving becomes a shared endeavor rather than an isolated burden, reducing crisis-driven hospital admissions and fostering greater confidence in home-based care.

Beyond caregiving tasks, communities play a crucial social and cultural role. Experiences of illness and dying are shaped by shared beliefs, norms, and rituals, which professional services cannot fully replicate. Community actors—such as faith leaders, cultural mediators, or trained community connectors—are often better positioned to address stigma, facilitate conversations about death, and support advance care planning in culturally meaningful ways. By acting as ethical and cultural mediators, communities help ensure that PC respects diversity and avoids imposing one-size-fits-all models that may inadvertently exclude marginalized groups.

The concept of “compassionate communities” encapsulates this broader vision. It reframes care for the dying as a collective social responsibility, integrating health services with schools, workplaces, local organizations, and civic structures (18, 40). In this perspective, PC is not confined to the final days or hours of life but becomes part of a community's caring capacity across the life course. Such an approach challenges the sharp boundary between professional and lay care, inviting health services to adopt roles of facilitation, education, and support rather than control. Specialists do not disappear in this model; rather, their expertise is redistributed through mentoring, shared decision-making, and responsive backup to community-based care.

In this sense, several community initiatives have emerged in different countries with the aim of promoting greater openness to dialogue about death and dying. Community initiatives such as death cafes, festivals, awareness weeks, books and public campaigns have emerged as ways to stimulate social dialogue and promote greater community literacy on the subject (41, 42). At the same time, volunteering has assumed a relevant role in this context, strengthening community support networks.

One of the emerging strategies in this context is the health navigation model, which aims to help patients and families understand and use the wider care system. In this model, navigators provide information, guidance on available resources and services, and facilitate access to community support. In addition, they are trained to address sensitive topics (such as palliative care, advance care planning, death and grief), they contribute to strengthening health literacy and promote a culture of care and support. This approach also seeks to involve populations at greater risk of health inequalities, such as people with low social support or in disadvantaged socioeconomic situations (43).

However, positioning communities as care partners is not without risks. There is a real danger that community engagement may be used rhetorically to justify the withdrawal of state responsibility or to normalize excessive caregiver burden, particularly for women and socially disadvantaged groups. A genuine paradigm shift therefore requires ethical safeguards, investment in caregiver support and respite, access to training and advice, and policies that recognize informal care as a public good rather than a private obligation (19). Communities cannot be empowered without resources, and partnership without power quickly becomes exploitation.

Crucially, community partnership also requires a cultural shift within health systems. Professionals trained in hospital settings may struggle to relinquish control or to recognize non-clinical forms of expertise. Valuing community knowledge challenges entrenched hierarchies and invites a more humble, relational form of practice. Trust, mutual respect, and continuity become central metrics of quality, alongside symptom control and service utilization.

In reshaping the PC paradigm, the move from hospital to community is ultimately about reclaiming care as a social practice. Communities do not replace hospitals; they complete them. When communities are acknowledged as care partners, PC can move closer to its ethical foundation: relieving suffering while honoring personhood, relationships, and belonging. The question is no longer whether communities should be involved, but whether health systems are willing to change deeply enough to make that partnership real.

Moreover, several structural and system-level challenges may hinder the shift of PC into the community (44). Workforce shortages—particularly of trained palliative care specialists, community nurses, and primary care providers—can constrain the delivery of timely, high-quality care outside hospital settings, while significant regional inequalities imply that rural or socioeconomically disadvantaged areas often face reduced access to services and infrastructure (45). At the same time, the increased reliance on informal care networks can intensify caregiver burden, placing emotional, physical, and financial strain on families and potentially compromising care sustainability (46). Resource constraints within health systems, including limited funding for community services, equipment, and coordination mechanisms, further restrict the scalability of these models. Additionally, variability in primary care capacity—such as differences in expertise, availability, and engagement with PC—can lead to inconsistent quality and continuity of care, undermining efforts to provide equitable, person-centered support in community settings.

## 5 Policy and system implications

International policy frameworks increasingly endorse community-based PC as an ethical and practical imperative. The World Health Organization frames PC as a core component of primary health care and universal health coverage, explicitly arguing that most PC should be delivered in the community, with specialist services providing backup rather than default care (7). Recent global analyses reinforce this position, emphasizing that integration across levels of care, sustainable financing, workforce development, and context-sensitive implementation are essential to avoid fragmentation. Without system-level alignment, community-based initiatives risk remaining pilot projects rather than transformative change (6, 26).

Most health systems retain hospitals as the primary locus of expertise, investment, and decision-making. Funding flows, performance indicators, and professional hierarchies continue to reinforce this centrality. Community-based PC challenges this architecture by asserting that most care needs—especially those related to symptom management, psychosocial support, and continuity—are best met outside institutional walls. Moreover, community-centered PC models are increasingly recognized as essential for enhancing EoL support, as sustained engagement of community members across different stages of care is associated with significant improvements in patient and family outcomes (47–49). In this sense, transitional care interventions are recommended to improve quality of life and symptoms among seriously ill patients. Given the uniqueness of people living with serious illness, organizational and national policies must better support transitions across care settings. This requires recognizing individual identity, clarifying professional accountability, and ensuring emotional and practical support within care models that are both structured and explicitly grounded in the defense of human dignity (30, 50).

As populations age, many individuals live longer with multiple chronic conditions and increasing frailty, leading to complex health needs that cannot be adequately addressed solely by curative approaches (51). Multimorbidity often results in declining functional ability and increased symptom burden, while ageism can contribute to inequities in access to appropriate care and support. Importantly, ensuring dignity at the EoL requires person-centered care that respects autonomy, comfort, and social wellbeing (50). Together, these factors reinforce the need for integrated PC approaches that address not only physical symptoms but also the psychological, social, and ethical dimensions of aging and dying.

Policy implications therefore extend beyond PC itself. A meaningful shift between models requires rebalancing system priorities toward longitudinal, relationship-based care. This entails strengthening primary care, community nursing, and social care infrastructures, and considering these components not as adjuncts to hospitals but as foundational pillars of the health system. Without such structural reorientation, community PC risks remaining peripheral and chronically under-resourced. Moreover, financing mechanisms must evolve to support continuity rather than crises. This includes funding for proactive care planning, interdisciplinary collaboration, caregiver education, and rapid response in the community. Crucially, reform should not frame community care as a cost-saving alternative to hospital care, but as an essential investment in quality, equity, and system sustainability. Cost containment may be an outcome, but it should not be the primary driver.

In sum, implementing community-based PC requires coordinated policies, sustainable funding, skilled workforces, integrated services, digital infrastructure, and clear evaluation metrics—all grounded in a public health approach that prioritizes equity, inclusion, and community capacity building (44).

## 6 Conclusion

Moving PC from the hospital to the community represents a profound change in paradigm rather than a simple relocation of services. It challenges entrenched assumptions about where care should be provided, who holds expertise, and how responsibility is shared. This paradigm shift is not achieved by a single intervention but through a coordinated framework. Integrated care ensures system connectivity, public health approaches build awareness, compassionate communities mobilize social support, transitional care secures continuity, and person-centered care ensures that all efforts align with what matters most to individuals. The evidence base increasingly supports community-based approaches, but their success depends on integration, investment, and cultural change. A community-based paradigm must therefore be accompanied by policies that invest in caregiver support, death literacy, and social infrastructure, rather than treating communities as cost-saving substitutes for formal care. A reimagined PC paradigm must be clinically robust, socially grounded, and ethically attentive, recognizing that dying is not only a medical event but a relational and communal experience. Community PC is most effective when it is embedded within long-term relationships, particularly in primary care, where professionals are familiar with patients' histories, identities, and social contexts. Strengthening these relationships enables earlier engagement, anticipatory planning, and smoother transitions between levels of care settings, reducing crisis-driven hospitalization. Embracing this shift offers an opportunity to align care with people's values, strengthen health systems, and restore humanity to EoL.

## Data Availability

The original contributions presented in the study are included in the article/supplementary material, further inquiries can be directed to the corresponding author.
